# Allicin Alleviated LPS-Induced Mastitis via the TLR4/NF-κB Signaling Pathway in Bovine Mammary Epithelial Cells

**DOI:** 10.3390/ijms24043805

**Published:** 2023-02-14

**Authors:** Hao-Yu Che, Chang-Hai Zhou, Chen-Chen Lyu, Yu Meng, Yun-Tong He, Hao-Qi Wang, Hong-Yu Wu, Jia-Bao Zhang, Bao Yuan

**Affiliations:** Department of Laboratory Animals, College of Animal Sciences, Jilin University, Changchun 130062, China

**Keywords:** allicin, mastitis, MAC-T, anti-inflammation, NLRP3, NF-κB

## Abstract

Dairy farming is the most important economic activity in animal husbandry. Mastitis is the most common disease in dairy cattle and has a significant impact on milk quality and yield. The natural extract allicin, which is the main active ingredient of the sulfur-containing organic compounds in garlic, has anti-inflammatory, anticancer, antioxidant, and antibacterial properties; however, the specific mechanism underlying its effect on mastitis in dairy cows needs to be determined. Therefore, in this study, whether allicin can reduce lipopolysaccharide (LPS)-induced inflammation in the mammary epithelium of dairy cows was investigated. A cellular model of mammary inflammation was established by pretreating bovine mammary epithelial cells (MAC-T) with 10 µg/mL LPS, and the cultures were then treated with varying concentrations of allicin (0, 1, 2.5, 5, and 7.5 µM) added to the culture medium. MAC-T cells were examined using RT–qPCR and Western blotting to determine the effect of allicin. Subsequently, the level of phosphorylated nuclear factor kappa-B (NF-κB) was measured to further explore the mechanism underlying the effect of allicin on bovine mammary epithelial cell inflammation. Treatment with 2.5 µM allicin considerably decreased the LPS-induced increase in the levels of the inflammatory cytokines interleukin-1β (IL-1β), interleukin-6 (IL-6), interleukin-8 (IL-8), and tumor necrosis factor-α (TNF-α) and inhibited activation of the NOD-like receptor protein 3 (NLRP3) inflammasome in cow mammary epithelial cells. Further research revealed that allicin also inhibited the phosphorylation of inhibitors of nuclear factor kappa-B-α (IκB-α) and NF-κB p65. In mice, LPS-induced mastitis was also ameliorated by allicin. Therefore, we hypothesize that allicin alleviated LPS-induced inflammation in the mammary epithelial cells of cows probably by affecting the TLR4/NF-κB signaling pathway. Allicin will likely become an alternative to antibiotics for the treatment of mastitis in cows.

## 1. Introduction

In dairy farming, the occurrence of mastitis in dairy cows seriously affects milk quality and milk yield [1]. Pathogenic infections are the most likely causative agent of mastitis, and a variety of Gram-negative bacteria, including Escherichia coli, are responsible for mastitis. LPS is an inflammatory endocytic toxin that is found on the outer wall of Gram-negative bacteria [2]. Pathogenic microorganisms invade udder tissue through the papillary ducts and multiply rapidly, resulting in infection of the udder [3]. In addition, increased levels of inflammatory mediators in the blood and increases in the numbers of mast cells and macrophages lead to the production of a variety of proinflammatory cytokines that induce neutrophils to enter the udder tissue from the blood, exacerbating the inflammatory response [4]. Inflammatory reactions that are sustained for long periods can damage the mammary glands; in severe cases, this can lead to tissue necrosis and atrophy [5,6].

Antibiotics are the most effective treatment for mastitis in dairy cows; however, due to the overuse of antibiotics in recent years [7], which causes resistance in pathogenic bacteria [8,9], and because the presence of antibiotics can be harmful to humans [10], the use of natural and harmless antibiotic alternatives has become a hot research topic and area of public concern [11]. Numerous studies have shown that extracts of certain plants can be used as antibiotic substitutes [12,13], and even their byproducts can act as feed additives and antibiotic substitutes [14]. Allicin, an active sulfur substance [15] that is found in garlic, onion, and other Allium plants [16], has many pharmacological properties, such as its anti-inflammatory, anticancer, antioxidant, and antibacterial properties [17,18,19,20]. Shen N et al. found that LPS-induced acute lung injury in rats could be ameliorated by allicin treatment, which ameliorated LPS-induced sepsis [21]. The following year, Li CL et al. showed that the injection of allicin into diabetic mice could reduce inflammation and thus prevent diabetic macroangiopathy [22]. Additionally, Nan B et al. found that allicin improved the acrylamide-stimulated NLRP3 inflammasome in rat blastocytes and that the release of inflammatory factors was reduced; thus, allicin exerted a hepatoprotective effect to prevent acrylamide-induced liver injury [23]. The addition of allicin to the diet also improved the survival and growth of rhododendron larvae by promoting intestinal development, reducing inflammation, and stimulating their appetite [24]. To date, studies on allicin have focused on two areas: first, its use as a feed additive to enhance appetite, promote animal intestinal development, and improve body resistance; and second, its use as a therapeutic agent to reduce inflammation, exert broad-spectrum antibacterial effects, and inhibit tumor growth [17]. Although these studies demonstrate the multiple effects of allicin, it is unclear whether allicin is beneficial for treating LPS-induced mastitis in cattle. The purpose of this study was to determine whether allicin could be useful for treating MAC-T cells stimulated with LPS. We measured the expression levels of the IL-1β, IL-6, IL-8, TNF-α, NLRP3, and Toll-like receptor 4 (TLR4) genes by RT–qPCR and measured the expression levels of the IL-1β, IL-6, TNF-α, NLRP3, TLR4, p65, phospho-p65 (p-p65), IκB-α, and phospho-IκB-alpha (p-IκB-α) proteins by Western blotting. Specifically, the effect of allicin on the cytosolic inflammatory response in the mammary epithelial cells of cows was analyzed by assessing the TLR4/NF-κB pathway after LPS stimulation. After establishing a model of mouse mastitis, we investigated whether allicin can reduce the expression of inflammatory factors in the mouse mammary gland and thereby alleviate LPS-induced mastitis in mice [25,26,27].

## 2. Results

### 2.1. Effect of Allicin on Cell Viability and Inflammatory Cytokine Levels

**Figure 1:**
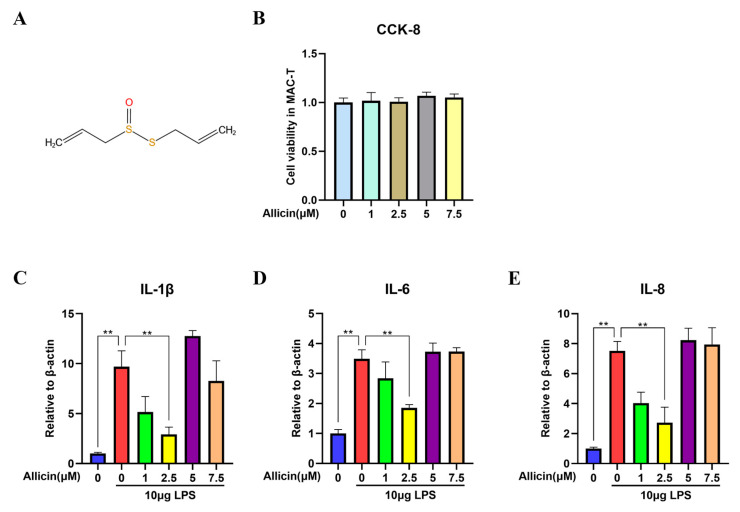
Effect of allicin on MAC-T cell viability and inflammatory cytokine levels in cells. (**A**) The structural formula of allicin. (**B**) Effect of allicin on cell viability. MAC-T cells were incubated with different concentrations of allicin (0, 1, 2.5, 5, and 7.5 µM) for 24 h. Cell viability was measured using the CCK-8 assay. (**C**–**E**) Effect of allicin on the expression levels of inflammatory cytokines in cells. The mRNA expression levels of IL-1β, IL-6, and IL-8 in MAC-T cells were measured using RT–qPCR with β-actin used as an endogenous control; **, *p* < 0.01.

To determine whether allicin would be harmful to cow mammary epithelial cells, a CCK-8 assay was used to assess the potential cytotoxic effects of allicin on cow mammary epithelial cells. Based on the results, the addition of different concentrations of allicin (1, 2.5, 5, and 7.5 µM) to the medium did not affect the viability of mammary epithelial cells from dairy cows (*p* > 0.05, Figure 1B). After determining the concentration at which allicin did not affect the viability of the cells, we determined whether allicin could inhibit LPS-induced inflammation by regulating inflammatory cytokine production. The levels of inflammatory cytokines such as IL-1β, IL-6, and IL-8 in mammary epithelial cells after the induction of inflammation were measured by RT–qPCR. As shown in Figure 1, LPS significantly increased the levels of IL-1β, IL-6, and IL-8 in the LPS group compared to the control group (*p* < 0.01). However, 1 µM and 2.5 µM allicin significantly inhibited inflammatory cytokine expression (*p* < 0.01, Figure 1A–C). Allicin had the most prominent effect at a concentration of 2.5 µM.

### 2.2. Effect of Allicin on LPS-Induced Activation of the NLRP3 Inflammasome

**Figure 2:**
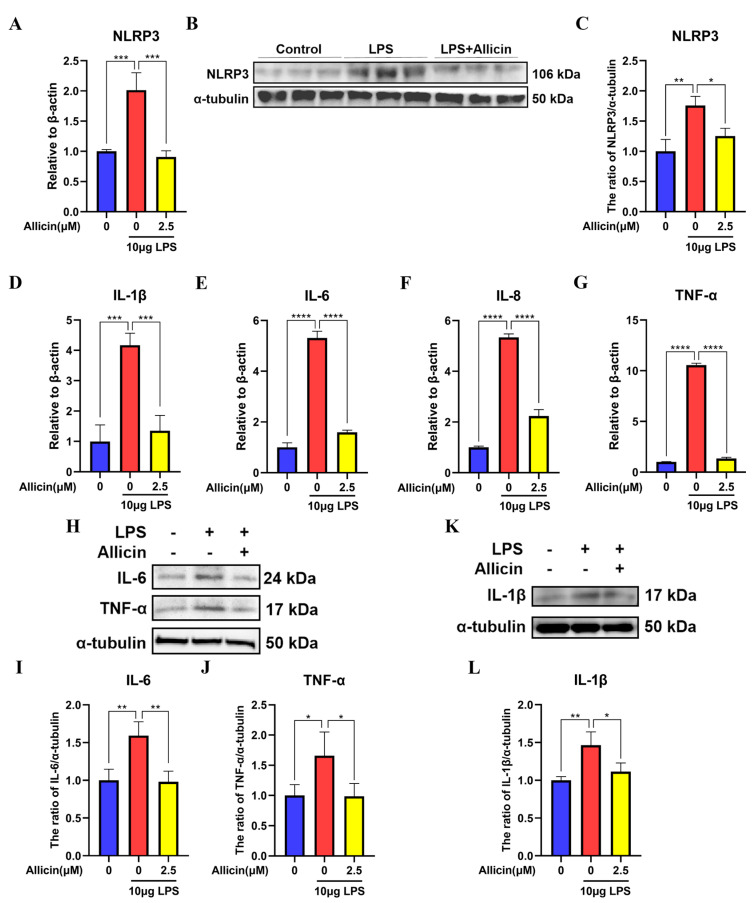
Effect of allicin on NLRP3 inflammasome activation in MAC-T cells. (**A**,**D**–**G**) The mRNA levels of NLRP3, IL-1β, IL-6, IL-8, and TNF-α in MAC-T cells were measured using RT–qPCR with β-actin used as an endogenous control. (**B**,**C**,**H**–**L**) The protein expression levels of NLRP3, IL-1β, IL-6, and TNF-α were detected using Western blotting, and alpha (α)-tubulin was used as a control. *, *p* < 0.05; **, *p* < 0.01; ***, *p* < 0.001; ****, *p* < 0.0001.

To determine the effects of allicin on the activation of the NLRP3 inflammasome, the mRNA expression of factors related to the NLRP3 inflammasome and inflammatory cytokines, such as IL-1β, IL-6, IL-8, and TNF-α, was measured by RT–qPCR, and the protein expression of NLRP3, IL-1β, IL-6, and TNF-α was measured by Western blotting. Real-time fluorescence quantification showed that the mRNA expression levels of NLRP3, IL-1β, IL-6, IL-8, and TNF-α were significantly higher in the LPS group than in the control group (Figure 2A,D–G), while 2.5 µM allicin significantly inhibited LPS-induced NLRP3, IL-1β, IL-6, IL-8, and TNF-α expression (Figure 2A,D–G). Western blotting showed significantly enhanced expression levels of NLRP3 (*p* < 0.01), IL-1β (*p* < 0.01), IL-6 (*p* < 0.01), and TNF-α (*p* < 0.05) compared to those in the control group. In addition, NLRP3 (*p* < 0.05), IL-1β (*p* < 0.05), IL-6 (*p* < 0.01), and TNF-α (*p* < 0.05) expression was significantly reduced in MAC-T cells treated with allicin (Figure 2B,C,H–L). These results suggest that LPS activated the NLRP3 inflammasome, while allicin inhibited this activity.

### 2.3. Effect of Allicin on TLR4 Expression

**Figure 3:**
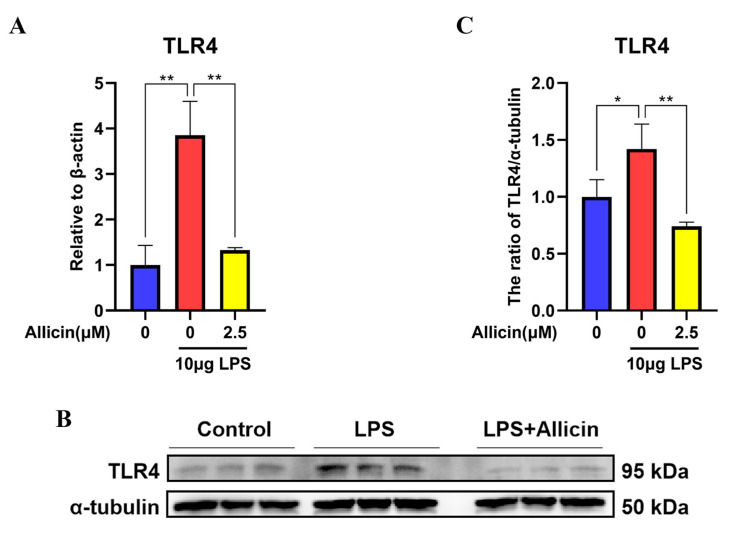
Effect of allicin on TLR4 expression in MAC-T cells. (**A**) The TLR4 mRNA level in MAC-T cells was detected using RT–qPCR with β-actin used as an endogenous control. (**B**,**C**) Expression levels of the TLR4 protein were detected using Western blotting, and α-tubulin was used as a control. *, *p* < 0.05; **, *p* < 0.01.

To elucidate whether allicin acts on TLR4 to affect the NF-κB signaling pathway, Western blotting and RT–qPCR were used to measure TLR4 expression. Real-time fluorescence quantification revealed significantly higher levels of TLR4 mRNA in the LPS group than in the control group (*p* < 0.01, Figure 3A), and the inhibitory effect of 2.5 µM allicin on LPS-induced TLR4 expression was significant (*p* < 0.01, Figure 3A). Western blotting also showed elevated TLR4 protein levels in the LPS group compared to the control group (*p* < 0.05, Figure 3B,C), while 2.5 µM allicin significantly decreased TLR4 expression (*p* < 0.01, Figure 3B,C).

### 2.4. Effect of Allicin on the NF-κB Signaling Pathway

**Figure 4:**
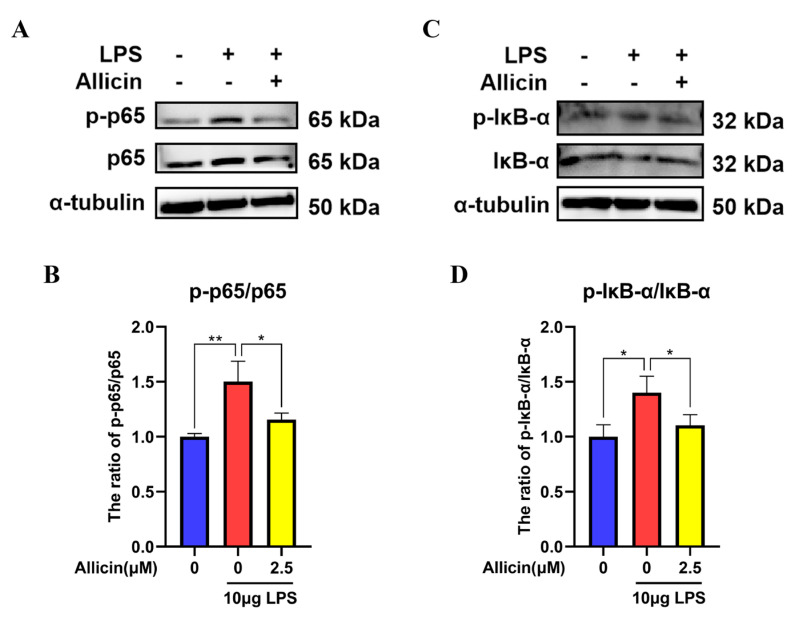
Effect of allicin on activation of the NF-κB signaling pathway in MAC-T cells. (**A**–**D**) Expression levels of p65 and IκB-α proteins were detected using Western blotting, and α-tubulin was used as a control. *, *p* < 0.05; **, *p* < 0.01.

Western blotting was used to measure the protein levels of NF-κB p65 and IκB-α to examine the effect of allicin on the NF-κB signaling pathway. Compared with the control, LPS treatment significantly increased the phosphorylation of p65 (*p* < 0.01, Figure 4A–D) and IκB-α (*p* < 0.05, Figure 4A–D). In contrast, the p65 and IκB-α phosphorylation levels were reduced by allicin (*p* < 0.05, Figure 4A–D).

### 2.5. Effect of Allicin on LPS-Induced Histopathological Changes

**Figure 5:**
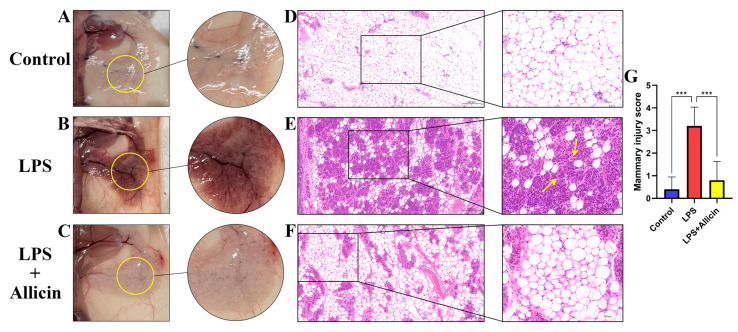
Effect of allicin on LPS-induced mammary gland injury. Histopathological changes in mammary tissue (H&E staining). (**A**–**C**) Photographs showing the morphology of mammary tissue in mice. (**D**–**F**) H&E staining of mouse mammary gland paraffin sections: yellow arrows indicate inflammatory cell infiltration in the mammary tissue. (**G**) Pathological damage scores for mouse mammary gland tissue. ***, *p* < 0.001.

By observing and staining breast tissue with H&E, the following experimental results regarding the effect of allicin on breast tissue injury were obtained. Visually, LPS significantly reddened and congested the breast tissues compared with the appearance of the control tissue (Figure 5A,B); despite this, allicin treatment significantly reduced breast injury (Figure 5C). From the perspective of pathological injury, a normal breast structure was observed in the control group, with no histopathological changes. A significant difference in mammary tissue structure was observed between the LPS group and the control group. The structure of the mammary tissue was disrupted by LPS treatment (*p* < 0.001, Figure 5D,E,G), as evidenced by increased thickness of the glandular follicular stroma, atrophy, or even necrosis; in addition, inflammatory cells had infiltrated the glandular follicles (Figure 5E). However, the damage to the mammary gland was substantially ameliorated after allicin treatment (*p* < 0.001, Figure 5F,G).

### 2.6. Effect of Allicin on the Level of Inflammation in an LPS-Induced Model of Mastitis In Vitro

**Figure 6:**
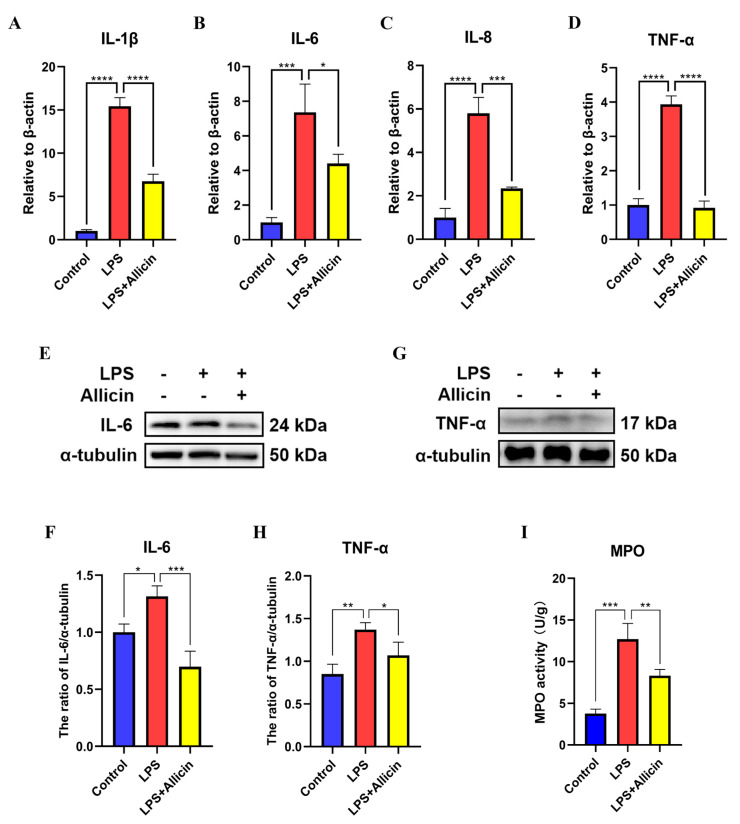
Effect of allicin on the expression of inflammatory cytokine genes in breast tissues. (**A**–**D**) mRNA expression levels of IL-1β, IL-6, IL-8, and TNF-α in breast tissues were detected using RT–qPCR with β-actin used as an endogenous control. (**E**–**H**) The protein expression levels of IL-6 and TNF-α in breast tissues were detected using Western blotting, and α-tubulin was used as a control. (**I**) Mammary gland MPO activity assay. *, *p* < 0.05; **, *p* < 0.01; ***, *p* < 0.001; ****, *p* < 0.0001.

To further confirm the effect of allicin on inflammation, inflammation-related indicators were investigated in vivo. The results of RT–qPCR and Western blotting revealed that LPS significantly increased the mRNA expression levels of the inflammatory cytokines IL-1β, IL-6, IL-8, and TNF-α compared with those of the control (Figure 6A–D), and LPS also significantly increased the protein levels of the inflammatory cytokines IL-6 and TNF-α (Figure 6E–H). Moreover, myeloperoxidase (MPO) activity was also elevated (Figure 6E–I), but these effects of LPS were eliminated by allicin treatment (Figure 6I). Based on these results, a model of mastitis was successfully established, and allicin alleviated mastitis by inhibiting inflammatory cytokine production.

## 3. Discussion

Typically, mastitis, which severely hinders dairy production, is caused by microbial infections that induce inflammation in the mammary gland [26]. One of the most common such pathogenic microorganisms is E coli, which is a major cause of severe mastitis [27]. Although antibiotics have been proven to be effective in treating mastitis, antibiotic residues left behind in milk and dairy products pose a health risk to humans. Thus, new antibiotic-free therapeutic strategies are needed to treat mastitis and replace antibiotics. An organosulfur compound named allicin is extracted from bulbs of garlic, which is a member of the Allium family with a variety of therapeutic effects, including antitumor and anti-inflammatory effects. Several recent studies have demonstrated that allicin could reduce renal ischemia in rats by inhibiting oxidative stress and inflammation [28]. Research on bovine mastitis has made considerable use of the LPS-induced mastitis model in mice as a practical approach to studying this condition [25]. To determine whether allicin inhibits LPS-mediated MAC-T cell inflammation, we investigated its anti-inflammatory properties and used mice to generate an in vivo model to elucidate the possible underlying mechanisms.

By histopathological examination, Xingchi Kan et al. [26] found that LPS caused severe damage to breast tissues, such as mammary glomerular damage and inflammatory cell infiltration, whereas the administration of pedunculoside improved these pathological changes, and according to the breast pathology in each group [29], allicin exerted a similar effect in this experiment. In the mouse model of LPS-induced mastitis, LPS caused severe damage to the mammary tissue and increased the secretion of inflammatory mediators in the mammary gland, but treatment with allicin significantly alleviated the mammary gland damage caused by LPS and reduced the secretion of inflammatory cytokines.

Inflammatory cytokines play a key role in host defense against invading pathogenic microorganisms [30]. However, excessive production of inflammatory cytokines can have a detrimental effect, and mastitis has been associated with a variety of inflammatory cytokines, including TNF-α, IL-6, IL-8, and IL-1β, which are considered to be essential inflammatory mediators that are involved in the development and progression of mastitis [31]. The production of IL-1β begins early in the course of infection and is considered to be one of the main mediators of inflammation [32]. Activated macrophages secrete TNF-α, which is a multifunctional proinflammatory cytokine that induces the production of other proinflammatory factors, such as IL-6, during the inflammatory response [33]. It is important for the immune system to produce adequate levels of proinflammatory cytokines, but excessive levels can cause serious cellular damage [34]. According to our findings, the mRNA expression levels of the inflammatory cytokines TNF-α, IL-6, IL-8, and IL-1β were significantly increased in MAC-T cells stimulated with LPS, but allicin attenuated the inflammatory response in LPS-stimulated MAC-T cells by significantly reducing the production of inflammatory cytokines.

NLRP3 inflammasome activation leads to inflammation [35], and its inactivation has been reported to help to alleviate inflammation in vitro and in vivo [36]. In addition, several studies have shown that in other cell types, such as Kupffer cells, the NLRP3 inflammasome could regulate the NF-κB signaling pathway, alleviating inflammation by reducing NLRP3 inflammasome secretion and inhibiting NF-κB activation [37]. In the current study, allicin inhibited NLRP3 inflammasome activation, decreased the protein expression of IL-1β, IL-6, and TNF-α, and decreased the release of the inflammatory factors IL-1β, IL-6, IL-8, and TNF-α. This further suggests that allicin can alleviate inflammation of the breast caused by LPS by reducing the production of inflammatory mediators.

Toll-like receptor signaling plays an essential role in immune responses to various intracellular pathogens [38]. TLR4 acts as an upstream receptor for NF-κB and specifically recognizes pathogen-associated molecules, such as LPS [39,40]. LPS has been reported to induce inflammatory responses through the TLR4/NF-κB pathway [41]. The NF-κB pathway is crucial for the expression of inflammatory cytokines [42,43,44]. NF-κB has been identified as a key nuclear transcription factor that plays a crucial role in regulating proinflammatory cytokine production [45]. NF-κB p65 is sequestered in the cytoplasm by binding the inhibitory protein IκB-α under basal conditions [46]. Different inflammatory conditions cause IκB-α to be phosphorylated and degraded, releasing the NF-κB p65 subunit and promoting the transcription of genes that are related to inflammation [47,48]. To obtain a deeper understanding of the mechanism by which allicin inhibits inflammatory responses in LPS-treated MAC-T cells, the effect of allicin on TLR4 expression was investigated. In the LPS-treated group, TLR4 expression was significantly increased, but allicin treatment decreased TLR4 expression. Allicin inhibited the phosphorylation of p65 and IκB-α in LPS-stimulated MAC-T cells. This suggests that allicin can inhibit the TLR4/NF-κB signaling pathway activated by LPS.

As a plant extract, allicin can significantly improve the production performance of livestock and poultry [49,50], enhancing the immune function of the livestock organism [51,52,53]. Allicin can play a function comparable to that of antibiotics [54], is less likely to produce drug resistance, and can also avoid problems such as drug residues with long-term rational use [55,56]. Allicin is expected to be an alternative to antibiotics in the treatment of mastitis in cows [57].

In conclusion, our results suggest that allicin inhibited activation of the TLR4/NF-κB signaling pathway and NLRP3 inflammasome and reduced the production of multiple inflammatory cytokines, thus exerting anti-inflammatory effects against the LPS-induced inflammation of MAC-T. The results further indicate that allicin attenuated the inflammatory response of the mammary gland in a mouse model of mastitis. This suggests that allicin has good protective effects against LPS-induced mastitis. Therefore, it is hoped that allicin can be used as a potential treatment for mastitis, replacing antibiotics and improving food safety and human health.

## 4. Materials and Methods

### 4.1. Animal Handling and Experimental Groups

This study was approved by the School of Animal Science of Jilin University and the Institutional Animal Care and Use Committee of Jilin University (license number: SY202206021). A total of 18 pregnant ICR mice aged 7 weeks (body weight of 35–40 g) were purchased from Changsheng Liaoning (Benxi, China). During the experiment, the mice were individually housed in cages in a room that was maintained at a temperature of 24 ± 1 °C and 65% humidity. Food and sterile water were provided ad libitum. Animal welfare laws, regulations, and ethical principles were strictly followed during all the study procedures. The mice were randomly assigned to three groups: the control group, the LPS group, and the allicin (2.5 mg/kg) + LPS group. Allicin was dissolved in dimethyl sulfoxide (DMSO) and diluted in sterile PBS to a final concentration of 2.5 mg/kg. In this experiment, lactating mice were anesthetized by intraperitoneal injection of sodium pentobarbital (40 mg/kg). Then, the proximal end of the nipple was removed. Syringes (100 µL) were used to inject LPS (10 mg/mL) through the mammary ducts and into the mammary glands (R4 and L4). The control mice received equal amounts of phosphate-buffered saline (PBS). Twenty-four hours after LPS injection, the allicin group received 3 intraperitoneal injections of 2.5 mg/kg allicin (every 6 h). As the final step, all the mice were anesthetized with sodium pentobarbital (40 mg/kg) and then inhaled CO_2_ for euthanasia. To preserve the mammary tissues, the tissues were stored at −80°C after harvest.

### 4.2. Cell Culture and Processing

An immortalized dairy cow mammary epithelial cell line (MAC-T cell line) was purchased from Qingqi Biotechnology Development Co., Ltd. (Shanghai, China). The cells were cultured in DMEM/F12 medium (BI, Kibbutz Beit Haemek, Kibbutz, Israel) supplemented with 10% fetal bovine serum (FBS) (LONSERA, Shanghai, China) and a 1% penicillin–streptomycin double-antibiotic solution (HyClone, South Logan, UT) in 25 cm^2^ cell culture flasks. The cells were cultured in cell culture flasks under a constant temperature of 37 °C and 5% CO_2_. When cell confluence reached approximately 80%, passaging or cellular planking was performed. In addition, a 1 mg/mL LPS stock solution was prepared by dissolving LPS (Sigma Aldrich, St. Louis, US) in sterile PBS. The final concentration of LPS used to treat the cells was 10 µg/mL. The LPS treatment time was 24 h. Allicin (20 mg) (Solarbio, Beijing, China) was dissolved in 1.2325 mL of dimethyl sulfoxide (DMSO, Sigma Aldrich) to generate a 100 mM concentrated stock solution of allicin. After the cell confluence reached 65%, LPS (0 or 10 µg/mL) was added to the medium, and the cells were incubated for 24 h. Then, allicin (0, 1, 2.5, 5, or 7.5 µM) was added, and the cells were incubated for another 24 h. The final concentration of allicin that was used for stimulation was 2.5 µM. At least three replicate wells were used for each experiment.

### 4.3. Cell Viability Assay

The effects of allicin on MAC-T cell viability were determined by CCK-8 assay. Each well of a 96-well plate was seeded with 100 µL of cell suspension, and both blank and control groups were established; the cells were precultured for 24 h. The original medium was discarded, and different concentrations of allicin were added to the new medium, after which the cells were incubated for another 24 h. Incubation continued for 1 h after the addition of 10 µL of CCK-8 solution (Beyotime, Shanghai, China) to each well. The absorbance at 450 nm was measured using an enzyme standard meter (Tecan, Safire, Austria). Three replicate wells were established for treatment with each of the concentrations of allicin.

### 4.4. RNA Extraction and RT–qPCR

Total RNA was extracted from cells using TRIzol reagent according to the manufacturer’s instructions (Invitrogen, Carlsbad, CA, USA). A NanoDrop2000 spectrophotometer (Thermo, Waltham, MA, USA) was used to measure the concentration of the extracted RNA on the basis of the OD ratio at 260/280 nm (OD 260/280). RNA samples with an OD ratio at 260/280 nm between 1.8 and 2.0 were used for subsequent experiments, and cDNA was synthesized using a reverse transcription kit (Monad, Wuhan, China). RT–qPCR was performed using MonAmp™ ChemoHS qPCR Mix (SYBR Green; Monad) and a CFX96 real-time PCR system (Bio-Rad, Hercules, CA, USA). The expression of all the target genes was measured with the endogenous gene β-actin as a control. The 2^−ΔΔCT^ method was used to calculate the relative expression of the target genes. The primer sequences are shown in Table S1.

### 4.5. Western Blotting

The cells were first cultured and treated with drugs in six-well plates, and lysates were prepared using radioimmunoprecipitation assay (RIPA) lysis buffer (Solarbio, Beijing, China) supplemented with the trypsin inhibitor phenylmethylsulfonyl fluoride (PMSF) (100:1 ratio) to extract the total proteins from the cells. The concentration of the extracted protein samples was determined by using the Thomas Brilliant Blue method. The total proteins were separated by 10% and 12.5% SDS–PAGE (EpiZyme, Shanghai, China). Then, the proteins were transferred to PVDF membranes (Sigma Aldrich, St. Louis, MO, USA). The PVDF membranes were blocked at room temperature for 1 h using 5% bovine serum albumin (BSA) (Sigma Aldrich, St. Louis, MO, USA) and TBST buffer (Solarbio, Beijing, China). Primary antibodies against IL-6 (CST, 12912, Danvers, MA, USA), TNF-α (CST, 11948, Danvers, MA, USA), IL-1β (Wanleibio, WL00891, Shenyang, China), NLRP3 (Wanleibio, WL02635, Shenyang, China), TLR4 (Wanleibio, WL00196, Shenyang, China), IκB-α (Wanleibio, WL01936, Shenyang, China), P-IκB-α (Wanleibio, WL02495, Shenyang, China), P65 (CST, 8242, Danvers, US), P-P65 (Wanleibio, WL02169, Shenyang, China), and α-tubulin (Abcam, ab40764, Cambridge, UK) were diluted 1:1000. The PVDF membranes were incubated overnight with these primary antibodies in a 4 °C refrigerator. The next day, the PVDF membranes were washed 3 times with TBST buffer for 7 min each. The secondary antibody (goat anti-rabbit IgG, Abcam) was diluted 1:4000 with blocking solution and incubated with the PVDF membranes at room temperature for 1 h. Then, the PVDF membranes were washed three times again with TBST buffer. Finally, the protein bands were visualized using a chemiluminescent substrate (Tanon, Shanghai, China), and the intensities of the bands were quantified using the ImageJ program.

### 4.6. Histopathological Analysis

Mice were euthanized for histological analysis of the mammary gland, and the mammary tissues were excised, fixed in 4% paraformaldehyde, and embedded in paraffin. Sections (4 µm) were stained with hematoxylin and eosin (H&E) and observed under a light microscope (Nikon, Eclipse CI, Japan). The sections were then scanned in a pathology section scanner (scanner model: Pannoramic SCAN, manufacturer: 3D HISTECH, country: Hungary) and viewed using viewing software (SlideViewer 2.5.0.143918) to assess pathological changes [26]. According to the degree of mammary interstitial edema of the mammary glands, the integrity of the acinar environment, and the infiltration of inflammatory cells in the acinar environment, the damage to the mammary tissue was scored as follows: 0, no injury; 1, mild injury; 2, moderate injury; 3, severe injury; 4, extreme injury.

### 4.7. MPO Activity Determination

MPO activity was used to measure neutrophil infiltration into breast tissues. According to the manufacturer’s instructions (Nanjing Jiancheng Company, Nanjing, China), breast tissues were homogenized in reaction buffer (w/v 1/9), and MPO activity was measured with an MPO activity assay kit.

### 4.8. Statistical Analysis

GraphPad Prism software (Windows version 8.02; GraphPad Software, Inc., San Diego, CA, USA) was used to analyze data using one-way or two-way analysis of variance (ANOVA); *p* values < 0.05 were considered to indicate statistically significant differences.

## Data Availability

Not applicable.

## Supplementary Materials

The following supporting information can be downloaded at: https://www.mdpi.com/article/10.3390/ijms24043805/s1.
